# Modern Microscopic Approaches to Astrocytes

**DOI:** 10.3390/ijms24065883

**Published:** 2023-03-20

**Authors:** Mitsuhiro Morita

**Affiliations:** Department of Biology, Graduate School of Sciences, Kobe University, Kobe 657-8501, Japan; mmorita@boar.kobe-u.ac.jp

**Keywords:** astrocyte, microscopy, electron microscopy, calcium imaging, optogenetics, Raman spectrum

## Abstract

Microscopy started as the histological analysis based on intrinsic optical properties of tissues such as the refractive index and light absorption, and is expanding to include the visualization of organelles by chemical staining, localization of molecules by immunostaining, physiological measurements such as Ca^2+^ imaging, functional manipulation by optogenetics, and comprehensive analysis of chemical composition by Raman spectra. The microscope is one of the most important tools in neuroscience, which aims to reveal the complex intercellular communications underlying brain function and pathology. Many aspects of astrocytes, including the structures of their fine processes and physiological activities in concert with neurons and blood vessels, were revealed in the course of innovations in modern microscopy. The evolution of modern microscopy is a consequence of breakthroughs in spatiotemporal resolutions and expansions in molecular and physiological targets due to the progress in optics and information technology, as well as the inventions of probes using organic chemistry and molecular biology. This review overviews the modern microscopic approach to astrocytes.

## 1. Introduction

Microscopy in life sciences addresses structures in living organisms and distributions of biomolecules, as well as measures and manipulates physiological phenomena. The astrocyte polarity between neurons and blood vessels, and gliotransmission, would not have been revealed without the innovations in modern microscopy. Modern microscopic approaches to astrocytes are challenges of optical visualization and manipulation of intricate astrocytes deep in the brain tissue, and their progress is attributed to breakthroughs in optics, information technology, chemistry, and molecular biology. This review overviews astrocyte research from a microscopic perspective and discusses future prospects.

## 2. Structural Studies of Astrocytes by Modern Microscopy

### 2.1. Discovery of Astrocytes

The history of optics can be traced back to ancient times. The word “lens” was derived from a Mesopotamian bean, lentil (English lentil, Latin lens), and convex lenses made of polished quartz crystal have been found in ancient Egyptian ruins, indicating that lenses were used as magnifiers or igniters in ancient times. The scientific revolution in the 17th century proposed in “The Origin of Modern Science (1949)” by Herbert Butterfield is closely associated with the dramatic advances in optics. The astronomical telescope, which was created during this period, brought about the geocentric view of the universe, and the microscope brought the cell-based view of living creatures. The compound microscope, consisting of objective and ocular lens, was invented by Hans and Zacharias Janssen in 1590, and their observation of plant tissue gave rise to the term “cell” (Micrographia, 1665 Robert Hook). Early microscopic observations were based on the contrast due to the light absorption of dense organelles or the light scattering by the cell wall or membrane. Eventually, the staining methods using chemicals that accumulate in organelles were developed to delineate the distribution and structure of neurons. The advance of microscopy to the discovery of astrocytes is described in detail in a review by Chvatal et al. [1]. In 1858, Rodolf Virchow proposed the concept of “neuroglia” as a filling substance between neurons in a lecture at the Berlin Medical University. Once Muller glial cells in the retina and Bergman glial cells in the cerebellum have been found as the cellular components of neuroglia, the identification of neuroglia in various brain regions was actively pursued, and in 1893 Michael Von Lenhossek coined the term “astrocyte” to refer to all of these cells. Santiago Ramón y Cajal sketched the diverse structures of astrocytes by using the Golgi staining of his modification and summarized them in his textbook [2]. This diversity was recently attributed to the change in astrocyte gene expressions due to the astrocyte–neuron interaction [3].

### 2.2. Electron Microscopic Analysis of Astrocytes

In the 20th century, new microscopic techniques such as electron microscopy and fluorescence microscopy were invented and revealed the common aspects of astrocytes including polarity and domain structure [4]. An astrocyte has two distinct processes, end-feet with blood vessels and perisynaptic astrocytic processes (PAPs) that interact with synapses and are polarized between blood vessels and neurons. These processes rarely overlap with neighboring astrocytes and occupy exclusive areas to form astrocyte domains in the brain. The invention of the transmission electron microscope (TEM) by Max Knell and Ernst Ruska in 1931 and following electron microscopic studies on cellular architecture in the mammalian central nervous system revealed the astrocytic structures surrounding blood vessels or neuronal processes [5], and in particular, the frequent contacts between synapses and astrocytic fine processes [6]. These early descriptions of astrocytic end-feet and PAPs have now evolved into the concepts of the blood–brain barrier and tripartite synapse.

**Figure 1:**
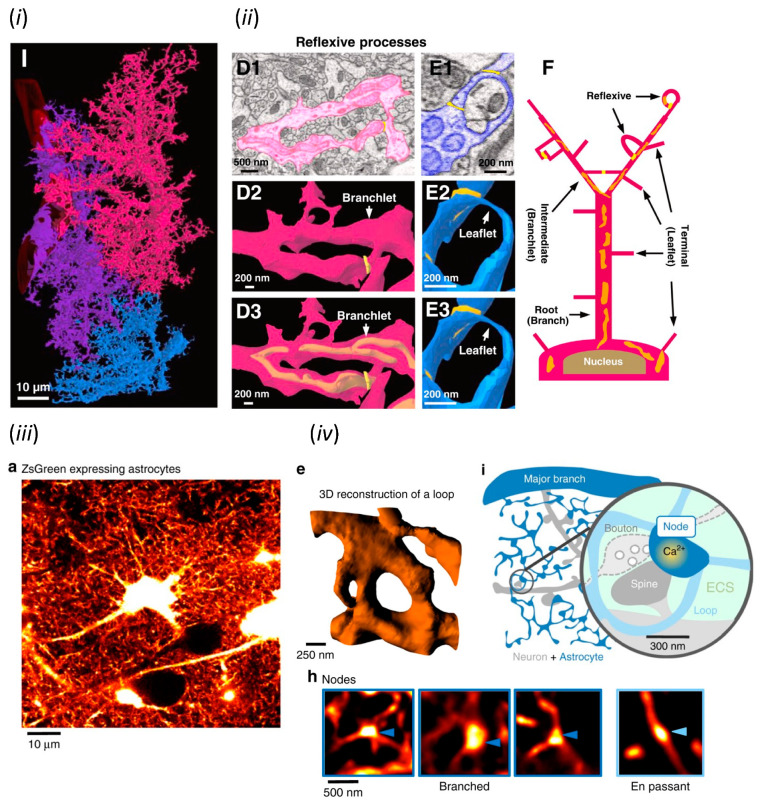
Structural studies of astrocytes by modern microscopy. (***i***) Astrocytic domain and (***ii***) reflexive process visualized by SBF-SEM. (***i***) and (***ii****)* were adapted with permission from Figures 1 and 2 in Aten et al., 2022 [7], respectively. (***iii***) In vivo astrocytes and (***iv***) their fine processes including node and sponge-like form visualized by STED microscopy. (***iii***), (***iv***) e and (***iv***) h were adapted with permission from Figure 1, while (***iv***) i was from Figure 8 in Arizono et al., 2020 [9], respectively.

Astrocytic processes are widespread nanoscale structures which are difficult to identify with light microscopy and were also difficult to be imaged in their entirety by electron microscopy for a long time after their discovery. However, the invention of serial block-face scanning electron microscopy (SBF-SEM) is revealing the full structure of astrocytic processes [7]. SBF-SEM automatically repeats surface sectioning by ultramicrotome and surface imaging by SEM of resin-embedded biological specimens, and visualizes a several hundred micrometers thick sample as a reconstructed digital three-dimensional image equivalent to several hundred to thousand consecutive TEM images. This technique has been realized by the improved sensitivity of backscattered electron scans due to a high-density heavy metal staining of cell membranes and the precise repeat of sectioning with the diamond knife in vacuum SEM chamber [8]. Three adjacent astrocytes in a 54.02 × 96.47 × 37.5 µm (X, Y, Z) region of the mouse hippocampal striatum radiatum have been three-dimensionally reconstructed from 500 consecutive images acquired by SBF-SEM (Figure 1i) [7]. Their processes not only invade between neurons and contact with synapses, but also form ring-like curved structures named the “reflexive process” and wrap around dendrites and axons (Figure 1ii). Thus, the astrocytic domain has been proposed as a spongiform structure evolving from the reflexive/leaflet processes in which the processes are closed, rather than a structure of divergently branching processes. 

### 2.3. Analysis of Astrocytic Nanoscale Structures by Light Microscopy

The function of astrocytes cannot be fully understood without the analysis of structures and motility of unfixed astrocytic processes. However, PAPs are nanoscale structures, which is difficult to visualize by conventional light microscopy, due to diffraction limits. The first imaging of unfixed PAPs has been achieved by stimulated emission depletion (STED) microscopy [9], a type of super-resolution microscopy, and the quantitative analysis of PAP dynamics by fluorescence recovery after photobleaching (FRAP) [10], respectively. STED microscopy limits the fluorescent region narrower than the diffraction limit by deactivating excited fluorophores by the irradiation of a doughnut-shaped de-activation spot created by spatial light modulator (SLM) [11]. Nanoscale structures of fluorescent protein (FP)-labeled PAPs and synapses in slice and in vivo preparations have been visualized and quantitatively analyzed by STED microscopy [9]. The loop-like structure of PAPs, which is equivalent to the reflexive process found by SBF-SEM [7], has been confirmed as a spongiform structure by STED microscopy (Figure 1iii). In addition, it has been found that the bulbous enlargements of PAPs called nodes contact with synapses and assemble along thin astrocytic processes, or shafts (Figure 1iv). Simultaneous Ca^2+^ imaging and superimposing Ca^2+^ elevations with STED images have shown the nodes as starting points of Ca^2+^ elevations. Since STED microscopy requires long dwell times during scanning for obtaining high resolution images, it is not suitable for imaging rapid changes of PAPs. Thus, the dynamics of PAP structure has been quantitatively analyzed by FRAP, which measures the time course of fluorescence recovery after intense light irradiation for bleaching fluorescent molecules. FRAP quantifies the diffusion process of fluorescent molecules, thus the recovery delays in relation to volume reduction. The regression of PAPs in association with synaptic plasticity has been demonstrated by the delay of FRAP measured from astrocyte processes filled with fluorescent dye following LTP induction and glutamate spillover in brain slice [10].

### 2.4. Analysis of Astrocytic Domains by Multicolor Imaging

The distribution of astrocytes in brain tissue has been studied for understanding how these cells fill the brain tissue as neuroglia and form syncytium, which is defined as a cell cluster connected intracellularly by gap junctions. Dye injection into adjacent astrocytes has shown that these cells occupy an exclusive domain in the brain [12], but the diversity, development, and turnover of this domain structure are not fully understood. Brainbow transgene, which irreversibly expresses multiple FPs at diverse combinations by stochastic recombination, has been developed to label cells in a specific cell population with distinct fluorescent profiles, allowing detailed analysis of cellular interactions and lineages [13]. Chromatic multiphoton serial (ChroMS) microscopy, which is a method to image multicolor tissue samples by single scans, have been developed for comprehensive analysis of tissue samples labeled by Brainbow. This microscope reconstructs multicolor images by the synchronous irradiation of multiple lasers and the detection of fluorescence in distinct wavelength ranges with multiple photomultipliers (PMTs) [14]. A fate analysis of astrocyte progenitors by Brainbow transgene and ChroMS microscopy has revealed the process by which astrocytes derived from the same progenitor cells distribute in domains and acquire morphological diversity, as well as the turnover of astrocytes in the adult brain [15].

## 3. Physiological Studies of Astrocytes by Modern Microscopy

### 3.1. Ca^2+^ Imaging of Astrocytes

Ca^2+^ plays crucial roles in the regulation of diverse cellular functions, and intracellular Ca^2+^ elevation can be evaluated by electrophysiological measurement of calcium current, aequorin bioluminescence, calcium-dependent gene regulation, and fluorescence Ca^2+^ indicator. A series of fluorescent Ca^2+^ indicators such as Fura2 and Fluo4 have been developed by introducing the structure of Ca^2+^ chelator into fluorophore and the acetoxymethyl modification at their carboxylic acid residues in the chelating site has allowed irreversible cell loading [16]. Ca^2+^ imaging using these indicators with digital video cameras has revolutionized astrocyte research [17]. Starting from the discovery of intracellular Ca^2+^ elevation in a glioma-derived cell line following serotonin treatment [18], it has been shown that diverse molecules involved in intercellular communications, such as neurotransmitters, hormones, and inflammatory mediators induce astrocytic Ca^2+^ elevation [19]. These facts have led neuroscientists to believe that glial cells, including astrocytes, play not only passive roles such as filling and electrically insulating the space between neurons, but also dynamic roles based on intercellular communications. A substantial number of publications have shown astrocytic Ca^2+^ elevations associated with neural activities and blood flow; however, their consequences still largely remain to be determined [17].

**Figure 2:**
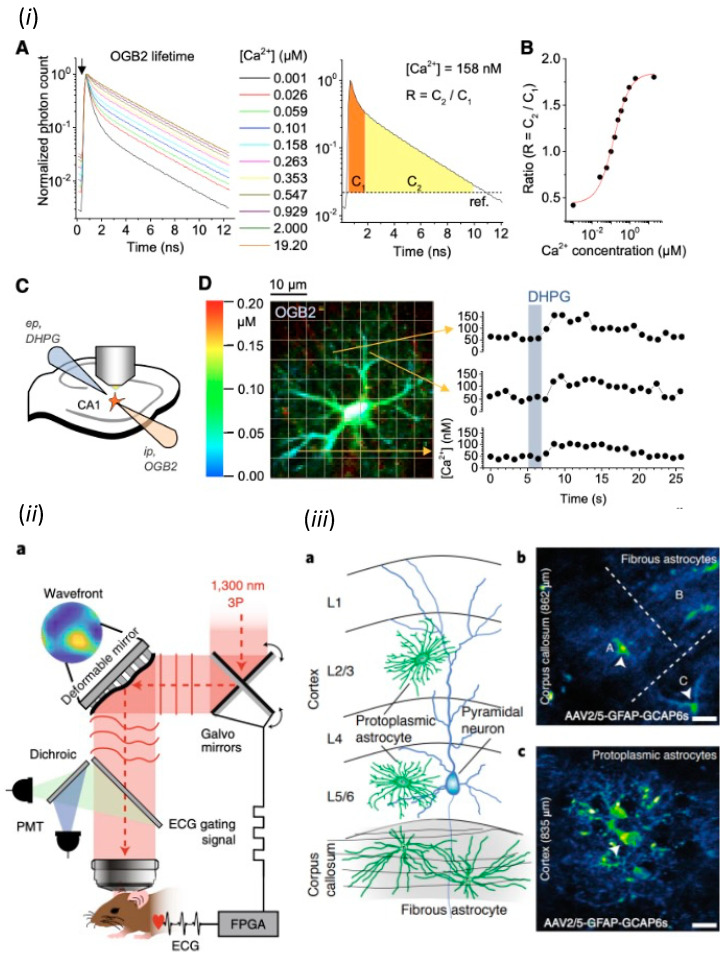
Physiological studies of astrocytes by modern microscopy. (***i***) Imaging of absolute Ca^2+^ concentration in in vivo astrocytes by 2P-FLIM. Adapted from Figure 1 in King et al., 2020 [22]. (***ii***) Three-photon microscopy with adaptive optics and (***iii***) its application for Ca^2+^ imaging of protoplasmic astrocytes in cerebral gray matter and fibrous astrocytes in white matter. (Scale bar, 20 μm). (***ii***) and (***iii***) were adapted with permission from Figures 1 and 4 in Streich et al., 2021 [32], respectively.

Some fluorescent Ca^2+^ indicators change fluorescence intensity and others change excitation or emission spectrum depending on Ca^2+^ concentration [16]. The former indicators represented by Fluo4 and GCaMP, can image cellular Ca^2+^ by time-lapse imaging using conventional image sensors, but their data do not directly reflect absolute Ca^2+^ concentration. In contrast, the latter represented by Fura2 and Cameleon can provide data reflecting absolute Ca^2+^ concentration by ratio imaging, which requires switching the excitation wavelengths or dual imaging after splitting fluorescence. However, ratio imaging is limited for high resolution microscopy, such as confocal laser scanning microscopy (CLSM) and two-photon excitation microscopy (2PM), because the focal position depends on wavelength. Thus, the invention of fluorescence lifetime imaging microscopy (FLIM) drastically changed the quantitative accuracy of Ca^2+^ imaging [20]. Since fluorophores have their intrinsic fluorescence lifetimes that are affected by environment, the fluorescence lifetimes of fluorescent Ca^2+^ indicators reflect absolute Ca^2+^ concentration. Thus, FLIM can measure absolute Ca^2+^ concentration by using existing fluorescent Ca^2+^ indicators and the results are not affected by the intracellular concentration and the fading of indicators. The fluorescence lifetime is also not affected by sample movement, because it is nanosecond order and measured in each pixel independently. In addition, the efficient calculation of absolute Ca^2+^ concentration and high-speed imaging at over 100 fps have been achieved by measuring fluorescence in time gates synchronized with pulsed lasers [20]. Furthermore, in vivo imaging of absolute Ca^2+^ concentration has been achieved by using two photon lasers for FLIM (2P-FLIM) [21]. 2P-FLIM have distinguished two astrocyte subpopulations of different resting Ca^2+^ concentrations in slice, as well as in vivo preparations [22] (Figure 2i). Another breakthrough achieved by FLIM is the imaging of fluorescent indicators of small fluorescence changes, especially those based on fluorescence resonance energy transfer (FRET) [23]. FLIM is now the gold standard for imaging fluorescent indicators that measure the conformational changes of proteins during metabolism and phosphorylation by FRET. The redox state and intracellular ATP concentration in astrocytes are imaged by FLIM imaging of FRET-based indicators [24]. FLIM also has allowed the chloride ion imaging of in vivo astrocytes with a classical synthetic chloride indicator, whose fluorescence is small and difficult to separate from tissue autofluorescence [25].

### 3.2. In Vivo Ca^2+^ Imaging of Astrocytes

The development of light microscopy in neuroscience has been aiming not only for higher resolution and sensitivity, but also for the expansion of the three-dimensional field of view (3D-FOV). Early Ca^2+^ imaging studies using conventional epifluorescence microscopy have characterized neurotransmitter responses [19] as well as Ca^2+^ dynamics commonly found in other non-excitable cells, including spontaneous Ca^2+^ elevation [26], Ca^2+^ oscillations [27], and Ca^2+^ waves [28] in cultured astrocytes. Then, CLSM allowed Ca^2+^ imaging of astrocytes within a few 10 μm from the surface of a brain slice, and demonstrated the astrocytic Ca^2+^ elevations associated with neural activities [29]. Furthermore, 2PM has allowed Ca^2+^ imaging of astrocytes within a few hundred micrometers from the cortical surface and demonstrated the astrocytic Ca^2+^ elevations associated with in vivo sensory stimulation [30]. Because of the three-dimensional complexity of astrocytes, three-dimensional scanning is shown to improve the quantitative aspect of 2PM Ca^2+^ imaging for Ca^2+^ elevations in processes of in vivo astrocytes [31]. Currently, the three-photon excitation combined with adaptive optics (Figure 2ii) have achieved structural analysis and Ca^2+^ imaging at depths of 1.4 mm below the cortical surface, i.e., beyond the cortex, the visualization of spines in the hippocampus, and the Ca^2+^ imaging of astrocytes in the white matter [32] (Figure 2iii). As tissue clearing techniques, such as CUBIC and SeeDB, remove lipid from cell membrane or replace solute with the solvent of the same refractive index as membrane lipid [33], the 3D-FOV is mainly limited by light scattering due to the difference in refractive index between water and membrane lipid. 2PM excites fluorophores in tissue with long wavelength lasers, which are less susceptible to scattering, and measure all fluorescence emitted by PMT. Therefore, scattering affects only the excitation process, and resolution is determined solely by the positioning of excitation light by scanner in 2PM. The scattering of excitation light can be further avoided by adaptive optics, in which the scattering of biological tissue is measured and then corrected by modulating the phase of light passing through the tissue [32,34]. The measurement of scattering uses fluorescent substances in tissues, such as FP-expressing cells [32] or fluorescent dye filled in blood vessels [34], as guide stars. Based on this information, the phase of light is designed to correct the scattering and is modulated either continuously by deformable mirrors or fragmented by liquid crystal spatial light modulators (LC-SLMs). A recently developed technique for expanding the correction of tissue scattering by using multiple guide stars [35] is expected to expand 3D-FOV further. In addition, the integration of this next-generation scattering correction technology in 2P-FLIM will allow imaging of absolute local Ca^2+^ concentrations in an expanded 3D-FOV of the brain.

Since PAPs are a nanoscale structure, they are rarely loaded with sufficient fluorescent Ca^2+^ indicator for Ca^2+^ imaging unaffected by background fluorescence and camera sensitivity. Thus, in vivo Ca^2+^ imaging of astrocytes has been primarily focused on cell bodies and stem processes. Although FLIM can image absolute Ca^2+^ concentration independently of the amount of Ca^2+^ indicator, an existing FLIM imaging study of astrocytic Ca^2+^ elevations placed region of interests (ROIs) on the cell body and stem process [22], presumably because the fluorescence of PAPs was below detectable level. Analysis of light-sheet fluorescence microscope images with spatiotemporal correlation screening allowed high contrast Ca^2+^ imaging; however, the Ca^2+^ elevation in PAPs is also not reported by this methodology, presumably due to insufficient fluorescence [36]. Ca^2+^ imaging of PAPs has been achieved not by microscope technology, but by membrane-anchored genetically encoded Ca^2+^ indicator (GECI), which distributes proportionally to the cell membrane. The fluorescence of PAPs was comparable to that of the cell body and stem processes, and unique Ca^2+^ dynamics of PAPs were characterized in two- photon imaging of in vivo astrocytes expressing membrane-anchored GECI [37]. PAPs lacked inositol triphosphate (IP3)-dependent Ca^2+^ release from endoplasmic reticulum, but showed microdomain Ca^2+^ transients, which is a Ca^2+^ efflux through the mitochondrial permeability transient pore. Future imaging of plasma membrane-bound GECI using 2P-FLIM will determine resting concentration and dynamics of Ca^2+^ in in vivo PAPs.

Internal brain structures such as the hippocampus, basal ganglia, and medial prefrontal cortex, which cannot be directly visualized through cranial windows, have functional association with behavior. It was shown that the astrocytic Ca^2+^ elevations in these regions are associated with psychiatric disorders [38,39,40] by the Ca^2+^ imaging of the internal brain structures of freely moving animals. For imaging the internal brain structures, the cerebral cortex is removed for direct access of conventional objective [38], or less invasively, a gradient-index (GRIN) lens of thin cylindrical and flat tip shape is inserted [39,40]. Two-photon imaging through a GRIN lens allowed Ca^2+^ imaging of large 3D-FOV in the internal brain structure [41]. Furthermore, a small head-mountable camera system equipped with a GRIN lens achieved Ca^2+^ imaging of the internal brain region of freely behaving mice [42]. Head-mounted microscopy systems equipped with three-photon excitation for deep brain imaging [43] or with a low-magnification objective for transcranial wide-field imaging of mice after skull clearing treatment [44] have been reported and they will be implemented for studying astrocytes in the near future.

Transcranial wide-field Ca^2+^ imaging was first reported by using a transgenic mouse line expressing high levels of GECI both in neurons and astrocytes and temporal skull clearing with a mixture of paraffin oil and glycerol [45]. This paper attributed the broad cerebral Ca^2+^ elevation following transcranial direct current stimulation to astrocytes by using both wide-field Ca^2+^ imaging at the tissue level and 2PM Ca^2+^ imaging at the cellular level. The recent progresses of wide-field Ca^2+^ imaging include the stable skull transparency by clearing with cyanoacrylate glue allowing long-term analysis and the optical compensation of hemoglobin absorbance [46,47], which interferes with GECI fluorescence signal, especially when GECI expression is not very high [48,49].

### 3.3. Optical Manipulations of Astrocytes

The optical manipulations of astrocytic Ca^2+^ elevations using caged compounds [50,51] or by optogenetics [52,53] are the major approaches to analyze the physiological and pathological implications of astrocytes in neural activities and blood flow. Caged Ca^2+^ or caged IP_3_ is uncaged by brief UV flashes [50] or localized scanning of two photon lasers [51], while opsins are activated by the irradiation or scanning of visible light [52,53]. Scanning is generally ineffective and inferior in temporal resolution, because confocal or two-photon laser spots are significantly smaller than cell volume and can activate a limited number of molecules. Single target stimulation with high spatial resolution has been achieved by focal brief irradiation through narrowed aperture [54],or localized laser scanning [51], while multiple target simulation by a digital micromirror device (DMD) [55] or computer generated holography (CGH) [56]. The DMD irradiates two-dimensional multiple spots with fast pattern control and little loss of light intensity [55]. Furthermore, three-dimensional multi-site random access photostimulation (3D-MAP), which irradiates three-dimensionally distributed spots with single-photon beam by modulating the angle of light to the DMD has been reported as a method for three-dimensional optogenetic stimulation by DMD [57]. On the other hand, CGH is a method for distributing three-dimensionally focused spots by phase modulation using SLMs [56]. Both 3D-MAP and CGH have the potential to stimulate non-targeted cells in light paths, but CGH using a two-photon laser can avoid this problem [58] and has already been used to evaluate functional connectivity between neurons in vivo [59]. The problem of CGH is the slow pattern control due to the incapability of SLMs to switch displayed patterns at high speed, but this will be sped up by displaying multiple patterns on the SLMs and switching between these patterns by controlling the light path to the SLM using a DMD. These next-generation optical methods will reveal the network of brain cells underlying brain function and pathology.

## 4. Imaging of Biomolecules and Water in Astrocytes by Modern Microscopy

Astrocytes are widely known to play crucial roles in regulating the brain environment by exchanging nutrients, waste, ions, neurotransmitters, and water. As a component of the blood–brain barrier, astrocytes are a major pathway for uptaking glucose and other metabolic substrates from the bloodstream to brain parenchyma, and synthesizing lipids from glucose for providing building blocks for neuronal processes and myelin [60]. Activity-dependent glucose uptake of the brain has been extensively analyzed by PET [61] and photoacoustic computed tomography [62] of 2-deoxyglucose analogues, and astrocyte uptake has also been imaged by in vivo two-photon microscopy of fluorescent 2-deoxyglucose analogue [63,64]. Genetically-encoded fluorescent indicators of glucose [65] and its metabolites [66] are now being reported, and they will soon visualize in vivo astrocyte uptake and metabolism of endogenous glucose by two-photon microscopes. Astrocytes also participate in intercellular communication in the brain by removing neurotransmitters and releasing gliotransmitters [67]. In addition, astrocytes highly express a water channel subtype, AQP4 at their vascular interface, and the deficiency of AQP4 causes the failure of the glymphatic system, which has been proposed as the integrated circulation of cerebrospinal and interstitial fluids [68]. Therefore, astrocytes play major roles in the dynamics of solutes as well as solvents in the brain. Thus, imaging of biomolecules and water in astrocytes is essential for understanding brain function and pathology, and is one of the future directions of microscopy.

Since glutamate transporters are electrogenic, the astrocytic glutamate uptake in hippocampal slices has been imaged by using voltage sensitive dye and photodiode array [69]. Similar voltage imaging has also detected the change of astrocytic membrane potential due to Na(+)/K(+) ATPase and potassium clearance by Kir4.1 [70]. ATP and its metabolite, adenosine, are the most widely accepted gliotransmitter, because luciferase-based luminescence imaging of ATP [71] and Ca^2+^ imaging-based fluorescent adenosine biosensor [72] have successfully imaged the releases of these substances from astrocytes in culture or in brain slices. Furthermore, in vivo astrocytic ATP release during inflammation has been imaged by 2PM and GFP-based biosensors expressed in astrocytes [73]. In order to validate the exocytosis of the gliotransmitter, the dynamics of intracellular vesicles in cultured astrocytes has been studied by total internal reflection fluorescence (TIRF) microscopy combined with uncaging of caged Ca^2+^ [74]; however, a recent electron microscopic analysis has shown that intracellular vesicles are rarely found in in vivo astrocytes [7].

**Figure 3:**
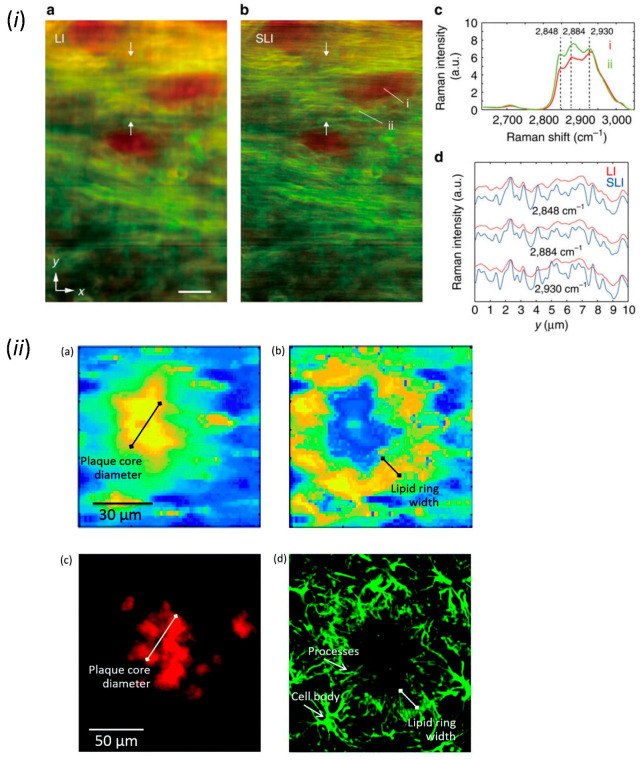
Imaging of biomolecules by Raman microscopy. (***i***) Raman spectrum imaging of beta sheets (red) and lipids (green) in the corpus callosum of fixed brain slice. (Scale bar, 20 μm). Adapted from Figure 4 Watanabe et al., 2015 [77]. (***ii***) Raman spectrum imaging of beta sheets in amyloid plaque and lipids in surrounding reactive astrocytes. Adapted with permission from Figure 4 in Palombo et al., 2018 [78].

The autofluorescence imaging of NAD(P)H in slices or in vivo by epifluorescence microscopy revealed the real-time influences of neural activities on the astrocytic energy metabolism [75]. Other non-fluorescent metabolites are able to be visualized by Raman microscopy [76]. This technique maps the spectrum of Raman scattering due to the intrinsic vibrations of molecular bonds in chemicals. Lipids in myelin [77] (Figure 3i), as well as beta sheets in beta-amyloid and lipids in reactive astrocytes [78] (Figure 3ii) in fixed brain tissues have been visualized by Raman microscopy. Furthermore, Raman microscopy can be used for the comprehensive analysis of biomolecules. The chemical composition of differentiating human iPS cells by Raman microscopy has found that glycogen is a novel neuronal differentiation marker of iPS cells [79]. In addition, Raman microscopy has been used to image water dynamics. Since the O-H vibration of H_2_O (water) is distinct from the O-D vibration in D_2_O (heavy water), the water dynamics have been visualized by using heavy water as a tracer in cell culture [80] and tissue preparation [81]. Thus, the improvement of sensitivity and resolution in Raman microscopy in the future is expected to allow the imaging of diverse biomolecules and water in astrocytes.

## 5. Conclusions

Modern microscopic approaches discussed in this review are summarized in Table 1. Many of these cutting-edge microscopes have been developed to achieve particular performances in laboratories, thus are not optimized for commercialization. In addition, most in vivo imaging methodologies require surgical treatments, which are accepted for rodents, but not humans, such as cranial window and skull clearing. Thus, they are not available for human study and diagnosis. Another important technical issue, which was little discussed in this review is data processing. Many innovations in resolution, field of view, and molecular and physiological targets were endorsed by the improvements of computer power and developments of machine-learning-based analysis. Since astrocytes have expanded during mammalian evolution, they must play crucial roles in the advanced information processing of our brain. Basic properties of neurons, such as action potentials, synaptic transmission, and ion channels are well conserved from the primitive nervous system of hydra to the human brain. On the other hand, neurons have become smaller, densely-populated, and complex both in structure and spike pattern during evolution. These neuronal changes were endorsed by the maintenance of electrical field, structure, nutrient supply, ionic environment, and signaling molecules by astrocytes. Thus, understanding the functions and pathological changes of astrocytes is one of the major challenges, and the microscopic approach to structures, activities, and biomolecules of small and densely-populated neurons together with astrocytes is the major experimental framework in neuroscience. Modern microscopy, which is expanding from traditional observation to functional imaging and manipulation, is evolving in a number of directions: spatial and temporal resolution, field of view and depth, and molecular and physical targets. As discussed in this review, the evolution of microscopic approaches is dependent not only on optics but also on the discovery and improvement of indicators and actuators. The current growing needs for diverse molecular and physical targets will be met by the collaboration between molecular biology and optics, such as FLIM and Raman microscopy. Once microscopy has evolved to image and manipulate neuronal activities together with astrocytic regulations of interstitial water, ions, nutrients, and signaling molecules, the data to model the information processing of our brain will be provided.

## Figures and Tables

**Table 1:** Modern microscopic approaches to astrocytes discussed in this review.

Category	Microscope	Achievements and Findings	References
Structure	Serial block-face scanning electron microscopy (SBF-SEM)	Reflexive process	Aten et al, 2022 [7]
	Stimulated emission depletion (STED) microscopy	Structure of living PAP	Arizono et al, 2020 [9]
	Fluorescence recovery after photobleaching (FRAP)	Dynamics of living PAP	Henneberger et al, 2020 [10]
	Confocal microscopy and dye injection	Astrocytic domain	Bushong et al, 2002 [12]
	Chromatic multiphoton serial (ChroMS) microscopy	Developmental process forming astrocytic domain	Clavreul et al, 2019 [15]
Physiology	Two photon fluorescence lifetime imaging microscopy (2P-FLIM)	Astocyte subpopulations of different resting Ca^2+^ level	King et al, 2020 [22]
		Redox state and ATP concentration	Köhler et al, 2023 [24]
		Cl^-^ imaging	Engels et al, 2021 [25]
	Three photon excitation with adaptive optics	Ca^2+^ imaging in subcortial structure	Streich et al, 2021 [31]
	Membrance-anchored GECI	Ca^2+^ imaging of PAP	Agarwal et al, 2013 [37]
	Two-photon endoscopy (GRIN lens)	Deep brain imaging (no astrocyte data)	Chien et al, 2021 [41]
	Head-mounted microscopy with three-photon excitation	Deep brain Ca^2+^ imagng of freely beehaving mouse (no astrocyte data)	Klioutchnikov et al, 2020 [43]
	Head-mounted microscopy with a low magnification objective	Wide field Ca^2+^ imaging of freely beehaving mouse (no astrocyte data)	Rynes et al, 2021 [44]
	Wide field Ca^2+^ imaging	Wide field Ca^2+^ imaging	Monai et al, 2016 [45]
	Furthermore, three-dimensional multi-site random access photostimulation (3D-MAP)	Target cell optogenetic stimulation (no astrocyte data)	Xue et al, 2022 [57]
	Two-photon Computer generated holography (CGH)	Target cell optogenetic stimulation (no astrocyte data)	Carmi et al, 2019 [58]
Biomolecule	GFP-based biosensor	In vivo ATP imaging	Wu et al, 2022 [73]
	Raman microscopy	Myelin imaging	Gomes et al, 2019 [76]
		Beta-amyloid imaging	Watanabe et al, 2015 [77]
		Water flow imaging	Yu et al, 2014 [81]
